# Simulation Study of cDNA Dataset to Investigate Possible Association of Differentially Expressed Genes of Human THP1-Monocytic Cells in Cancer Progression Affected by Bacterial Shiga Toxins

**DOI:** 10.3389/fmicb.2018.00380

**Published:** 2018-03-13

**Authors:** Syed A. Muhammad, Jinlei Guo, Thanh M. Nguyen, Xiaogang Wu, Baogang Bai, X. Frank Yang, Jake Y. Chen

**Affiliations:** ^1^Institute of Biopharmaceutical Informatics and Technologies, Wenzhou Medical University, Wenzhou, China; ^2^Wenzhou Medical University 1st Affiliated Hospital, Wenzhou, China; ^3^Institute of Molecular Biology and Biotechnology, Bahauddin Zakariya University, Multan, Pakistan; ^4^Department of Computer and Information Science, Purdue University Indianapolis, Indianapolis, IN, United States; ^5^Institute for Systems Biology, Seattle, WA, United States; ^6^Department of Microbiology and Immunology, Indiana University School of Medicine, Indianapolis, IN, United States; ^7^Informatics Institute, School of Medicine, The University of Alabama at Birmingham, Birmingham, AL, United States

**Keywords:** cDNA dataset, Shiga toxin, differential expression, cancers, enrichment analysis, TFBS

## Abstract

Shiga toxin (Stxs) is a family of structurally and functionally related bacterial cytotoxins produced by *Shigella dysenteriae* serotype 1 and shigatoxigenic group of *Escherichia coli* that cause shigellosis and hemorrhagic colitis, respectively. Until recently, it has been thought that Stxs only inhibits the protein synthesis and induces expression to a limited number of genes in host cells, but recent data showed that Stxs can trigger several signaling pathways in mammalian cells and activate cell cycle and apoptosis. To explore the changes in gene expression induced by Stxs that have been shown in other systems to correlate with cancer progression, we performed the simulated analysis of cDNA dataset and found differentially expressed genes (DEGs) of human THP1-monocytic cells treated with Stxs. In this study, the entire data (treated and untreated replicates) was analyzed by statistical algorithms implemented in Bioconductor packages. The output data was validated by the k-fold cross technique using generalized linear Gaussian models. A total of 50 DEGs were identified. 7 genes including TSLP, IL6, GBP1, CD274, TNFSF13B, OASL, and PNPLA3 were considerably (<0.00005) related to cancer proliferation. The functional enrichment analysis showed 6 down-regulated and 1 up-regulated genes. Among these DEGs, IL6 was associated with several cancers, especially with leukemia, lymphoma, lungs, liver and breast cancers. The predicted regulatory motifs of these genes include conserved RELA, STATI, IRFI, NF-kappaB, PEND, HLF, REL, CEBPA, DI_2, and NFKB1 transcription factor binding sites (TFBS) involved in the complex biological functions. Thus, our findings suggest that Stxs has the potential as a valuable tool for better understanding of treatment strategies for several cancers.

## Introduction

Shiga toxins (Stxs) are bacterial cytotoxins mainly produced by enteric pathogens. It is a family of related toxins with two major groups, Stx1 and Stx2, encoded by genes belonging to the lambdoid prophages genome (Friedman and Court, 2001). The sources of Stxs includes *Shigella dysenteriae*, the Shiga toxigenic group of *Escherichia coli* (STEC) including serotypes O157:H7, O104:H4, and other enterohemorrhagic *E. coli* (EHEC) (Beutin, 2006; Spears et al., 2006) responsible for millions of cases of severe dysentery, food poisoning, gastroenteritis and bowel necrosis (Mims et al., 1993; Vogt and Dippold, 2005; Todar, 2007; World Health Organization, 2016), and this situation is worse in underdeveloped countries.

The Stxs are capable to cross epithelial, endothelial, leukocytic, lymphoid and other neuronal cells through transcytotic or paracellular mechanisms (Malyukova et al., 2009), may then associate with blood monocytes, macrophages and neutrophils to circulate the bloodstream and bind to toxin-binding glycosphingolipid Gb3 receptor by clathrin-dependent or clathrin-independent mechanisms (Lingwood, 2003; Schweppe et al., 2008). After internalization, the toxins undergo retrograde intracellular flow to reach the endoplasmic reticulum (Sandvig et al., 2002). Importantly, the toxin receptor, Gb3, has a limited expression in normal tissues but is overexpressed in several types of cancer and besides apoptosis, may cause “apoptosis induced proliferation” (Torgersen et al., 2010). Stxs are cytotoxic proteins that enter the host cell via macropinosome and function as an N-glycosidase, cleave a specific adenine nucleobase from the 28S RNA of the 60S subunit of the ribosome, thereby halting host cell protein synthesis (Sandvig et al., 2010; Lukyanenko et al., 2011). This activity of the toxin resides in the A subunit and the pentamer of similar B subunit intercedes toxin binding to the Gb3 (Fraser et al., 1994; Lingwood et al., 2010; Melton-Celsa, 2014). These toxins can also activate host cell's signaling pathways and trigger apoptosis in many cell types. They induce apoptosis of epithelial, endothelial, leukocytic, lymphoid and neuronal cells (Tesh, 2010). After activation, the interleukin-1 (IL-1) and tumor necrosis factor alpha (TNF-α) sensitize endothelial cells to the action of Stx *in vitro* by increasing Gb3 expression (van de Kar et al., 1992; Stricklett et al., 2002). Upon sensitization, the innate immune response stimulated by Stxs may contribute to the development of vascular lesions (Ramegowda et al., 1999). It has been reported that when human monocytic THP-1 cells are treated with Stxs, they secrete tumor necrosis factor alpha (TNF-α)-, interleukin-1 (IL-1), and interleukin-6 (IL-6), which further alter the expression of these cytokines and chemokines (Leyva-Illades et al., 2010). Transcriptional regulation involves prolonged activation of stress-associated protein kinases JNK (c-Jun N-terminal kinases) and p38, extracellular signal-activated kinase1/2 (ERK1/2), and activation of transcription factors NF-kappaB (NF-κB) (nuclear factor) (Thorpe et al., 2001; Harrison et al., 2005). Recent evidence indicates that signaling through MAPK pathways and proapoptotic proteins -mostly caspases- can induce proliferation of neighboring surviving cells to replace apoptotic and dying cells. This “apoptosis induced proliferation” is critical for tissues regeneration and cancer progression (Tesh, 2010; Ryoo and Bergmann, 2012).

The expression profiling of treated and untreated THP-1 cells verifies that Stxs are responsible for genetic alterations (Leyva-Illades et al., 2010). Studies show that Stxs is associated with an elevated secretion of cytokines and other chemical mediators responsible for numerous diseases including cancer (DesRochers et al., 2015; Hattori et al., 2016). *E. coli* producing Stxs was isolated from cancer and diarrheagenic individuals (Chao et al., 2017). It has been observed that Stxs significantly caused increased expression of GRO, G-CSF, IL-1β, IL-8, and TNFα in THP-1 like cells with an increased level of pro-inflammatory cytokines and chemokines causing several diseases (Brandelli et al., 2015). The exact mechanism of influence of gene expression by Stxs as well as the functions of these influenced genes in respect to pathophysiology and molecular biology of cancer development have not been completely understood (Tesh, 2010). In this regard, cDNA microarray technology is a valuable tool to discover differential gene expression. The accumulated data from gene expression studies in public repositories provides an opportunity to construct pooled gene expression data sets from a larger number of individuals. In this study, we performed cDNA differential analysis of the publicly available dataset to discover the DEGs and to further explore the transcriptional responses of human THP1-monocytic cells affected by bacterial Stxs. We uncovered the aberrantly expressed genes which are significantly linked to cancer. We also report the analysis of the pathophysiological pathways and predicted potential regulatory motif of these genes.

## Materials and methods

### Accession of cDNA dataset

The goal of this study was to identify aberrantly expressed cancer-related genes in the human THP1-monocytic cells lines affected by bacterial Shiga toxins (Supplementary Figure 1). We acquired the cDNA microarray dataset (NCBI, 2015) GSE19315 (Leyva-Illades et al., 2010) from Gene Expression Omnibus database (http://www.ncbi.nlm.nih.gov/geo/query/acc.cgi?acc=GSE19315)^1^. This dataset examined the transcriptional changes in human THP-1 cells to Stxs toxins. It was performed by taking three untreated (controls) and six treated replicates (samples) followed by the real-time studies. The Stxs changed the genetic expression which is associated with the increased production of cytokines and other proinflammatory mediators. The GPL570 [HG-U133_Plus_2] Affymetrix Human Genome U133 Plus 2.0 Array (Affymetrix, Inc., Santa Clara, CA, 95051, USA, Technology: *in situ* oligonucleotide) platform was used, and the Annotation information (hgu133plus2) of probes was used to detect the gene expression.

### Normalization and identification of differentially expressed genes (DEGs)

Pheno-data files of this dataset were prepared in identifiable format and missing values imputed (Troyanskaya et al., 2001). The Bioconductor packages including ArrayQuality Metrics on R version 3.1.3 were used to execute the preprocessing steps of normalization and quality control (Bolstad et al., 2003; Fujita et al., 2006; Obenchain et al., 2014). Robust Multi-array Analysis (RMA) was used to adjust the background and normalization (Yoon et al., 2004) for perfect matches (PM) and mismatches (MM) in order to get a summary of intensities (summary statistic). To assess the quality of RNA in samples, AffyRNAdeg, summaryAffyRNAdeg, and plotAffyRNAdeg Bioconductor packages were used for degradation analysis (Affymetrix, 1999, 2001). After normalization, we performed the relative study and identified differentially expressed genes by pairwise comparison from genomic experiments (Tusher et al., 2001) and multiple testing corrections were completed by Benjamini-Hochberg method (Benjamini and Hochberg, 1995). The Bioconductor package LIMMA, a modified statistic that is proportional to the statistic with sample variance offsets, was used to shortlist the DEGs. By this package, duplicate spots and quality weights were measured. The moderated statistics were calculated; genes were ranked with respect to the resulting scores and *p*-values. A false discovery rate (FDR) less than 0.05, *p* ≤ 0.05, Average Expression Level (AEL) ≥40% and an absolute log fold change (logFC) greater than 1 were set as the significant cutoffs.

### K-fold cross validation

We used the k-fold technique (Seymour, 1993) to validate the shortlisted differentially expressed genes using the Bioconductor “boot” package (Ripley, 2010). Bootstrapping can be helpful to correct some of the bias associated with the analysis. The generalized linear models (Gaussian) were applied and the “cv.glm” function was used to assess the k-fold cross validation for these cases. This function, firstly, predicts the error of the raw cross-validation followed by the adjusted cross validation estimation. The Gaussian function was trailed by the complete Leave-One-Out-Cross-Validation (LOOCV) procedure. The LOOCV method is instinctively named as one case is left out as the testing set and the rest of the data are used as the training set (Ripley, 2010).

### Curation of cancer-related genes

From the shortlist of DEGs, cancer-associated genes were curated from OMIM (Online Mendelian Inheritance in Man) database (www.ncbi.nlm.nih.gov/omim)^2^ and Cancer GeneticsWeb database (www.cancer-genetics.org)^3^ to filter the significant cancer-related genes. The roles of sorted genes in cancer were evaluated through reported experimental studies using PubMed^4^ database.

### Cluster and functional enrichment analysis of DEGs

We performed the cluster analysis (Eisen et al., 1998) based on expression values in each sample to verify the variations in gene expression levels between untreated and treated replicates. The functional Annotation and pathway enrichment analysis of cancer-related genes help us to reveal biological functions (Nam and Kim, 2008; Muhammad et al., 2015), and it was performed using the web-based DAVID (Database for Annotation Visualization and Integrated Discovery) (Huang da et al., 2009) and FunRich Annotation tools (Pathan et al., 2015).

### Construction of protein-protein interaction (PPI) network

Proteins usually interact with each other to perform biological functions (Li et al., 2004; Muhammad et al., 2014; Naz et al., 2015). Therefore, the interacting partners of cancer-related genes were assessed with a high confidence score (0.999) from the STRING (Search Tool for the Retrieval of Interacting Genes/Proteins) (Szklarczyk et al., 2011) and HAPPI (Human Annotated and Predicted Protein Interaction) databases (Chen et al., 2009), and PPI-network were constructed using Cytoscape software (Cline et al., 2007) version 3.2.1. STRING and HAPPI connect major databases and predict interactions based on experiments, text mining and sequence homology. The role and association of these genes (source and target) in cancer were accessed from OMIM (www.omim.org), Cancer Genetics Web (www.cancerindex.org) and National Cancer Database (www.facs.org)^5^. The relationships between source proteins and target proteins (interacting proteins) were finally calculated.

### *De novo* prediction of regulatory motifs

To better understand the complex mechanisms of cancer-related, differentially expressed genes controlling important biological functions at transcriptional and post-transcriptional levels, oPOSSUM web tool (Ho Sui et al., 2005) was used to identify the transcription factor (TF) binding sites and over-represented regulatory motif of target matrices (Pavesi et al., 2004) in promoter regions of expressed genes using default parameters.

## Results

### Microarray analysis, normalization, and RNA degradation plots

**Figure 1:**
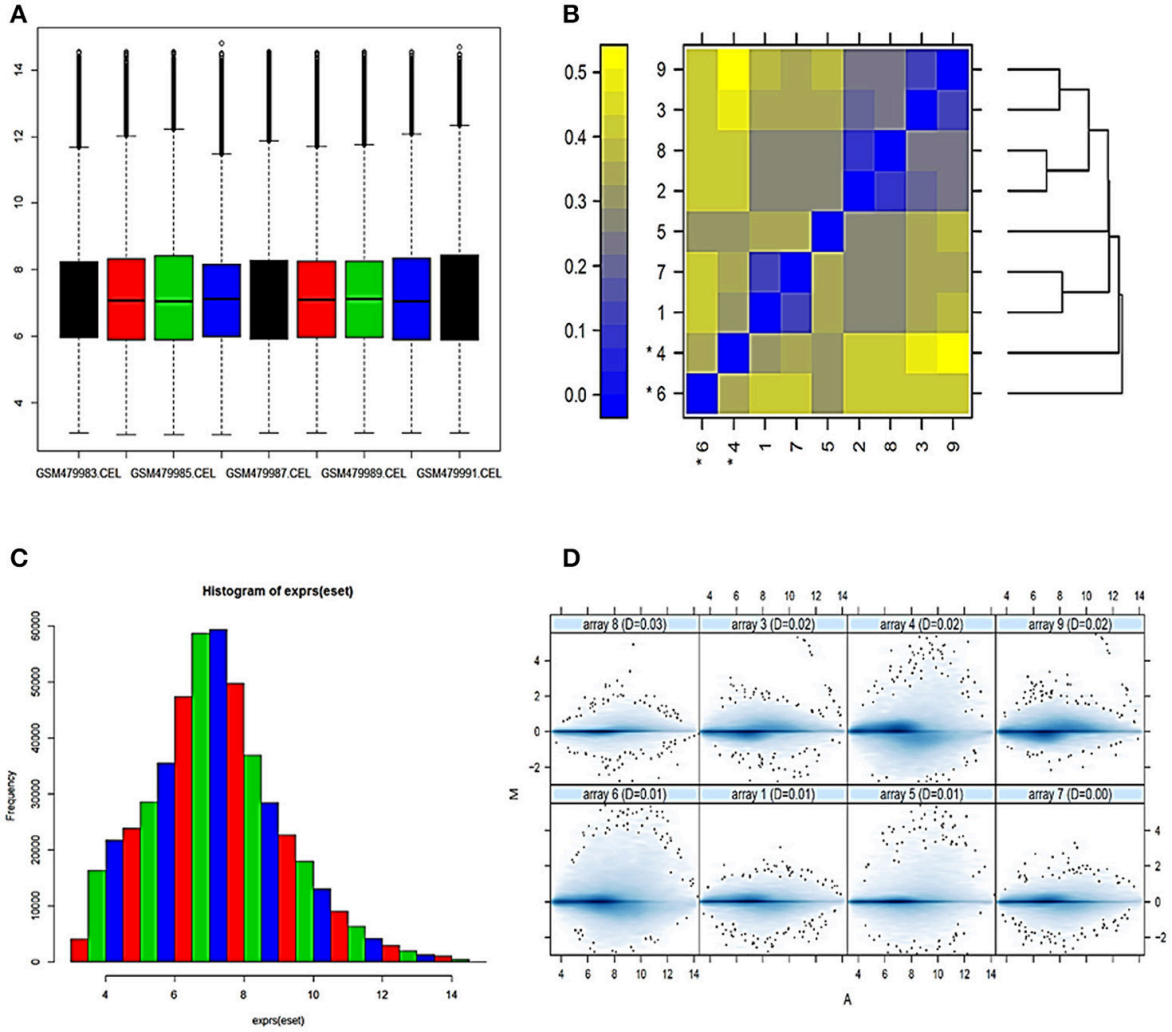
Normalization and analysis of array quality metrics **(A)** Box-plot representing summaries of the signal intensity distributions of the arrays. Each box corresponds to one array. Outlier detection was performed by computing the Kolmogorov-Smirnov statistic Ka between each array's distribution and the distribution of the pooled data **(B)** Heatmap of the distances between arrays. The color scale is chosen to cover the range of distances encountered in the dataset **(C)** Histogram representing expression after normalization **(D)** MA plots. M and A are defined as: M = log_2_(I_1_) – log_2_(I_2_), A = 1/2 (log_2_(I_1_)+log_2_(I_2_)), where I_1_ is the intensity of the array studied, and I_2_ is the intensity of a “pseudo”-array that consists of the median across arrays.

**Figure 2:**
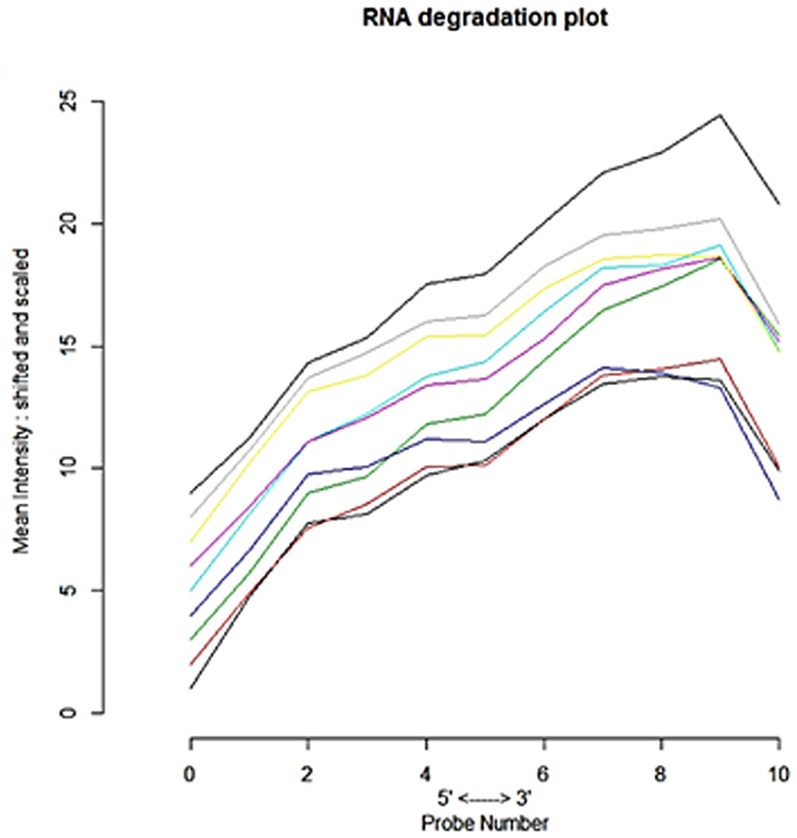
Side-by-side plot produced by plotAffyRNAdeg representing 5′-3′ trend to assess the severity of degradation of RNA and significance level.

The microarray data consists of 9-samples with 54675 probe ids from a study of the effect of bacterial Shiga toxins on human THP1 macrophage/monocytic cell lines (Leyva-Illades et al., 2010). The AffyBatch object comprises the size of the array 1,164 × 1,164 features with 54,675 affyids. Background correction and normalization by quantile normalization were completed to avoid systematic variation between samples. The normalized probe-level data represents expression levels of genes of entire DNA chip (Figure 1) involved in experimental condition and individual probes in a probe set were ordered by location relative to the 5′ end of the targeted RNA molecule. The 3′/5′ intensity gradient has been shown to depend on the degree of competitive binding of specific and of non-specific targets to a particular probe. Poor RNA quality is linked with a reduced amount of RNA quantity hybridized to the array paralleled by a declined total signal level. Increasing degrees of saturation reduce the 3′/5′ intensity gradient. The probe set contained the target gene at a position near the 3′ end of the corresponding transcripts. A side-by-side plot was produced by the function plotAffyRNAdeg (Figure 2), and the function summary of AffyRNAdeg produced a single summary statistic for each array in the batch (Table 1), presenting an assessment of the severity of RNA-degradation and significance level. We focused to sort out important factors in the cDNA dataset such as sequences biases, RNA degradation and quality of RNA to ensure the reliability of the dataset to identify transcriptional variations of the original samples. We standardized the sample handling procedures through normalization and assessed the optimal RNA reliability threshold using statistical and algorithm discrimination measures.

**Table 1:** A summary statistic for each array in the batch, assessing the severity of RNA degradation and significance level.

**S. no**	**Arrays/Samples**	**Slope**	***P*-value**
1	GSM479983.CEL	1.01000	0.00109
2	GSM479984.CEL	1.000000	0.000827
3	GSM479985.CEL	1.41e+00	1.15e-05
4	GSM479986.CEL	0.6570	0.0183
5	GSM479987.CEL	1.18000	0.00033
6	GSM479988.CEL	1.10e+00	9.65e-05
7	GSM479989.CEL	0.91700	0.00196
8	GSM479990.CEL	0.97500	0.00112
9	GSM479991.CEL	1.40e+00	1.32e-05

### Identifying differentially expressed genes (DEGs)

Our analyses revealed a total of 50 DEGs from Shiga effected human THP1-monocytic cells between 03 untreated and 06 treated to replicate samples, including 01 up-regulated (2%) and 49 down-regulated (98%) DEGs (Table 2). For generalized linear models, the “cv.glm” function estimated the k–fold cross-validation prediction error. The dispersion parameter for Gaussian is 0.03268, which indicates the confidence models. The similar delta value of 0.032715 with 10 K-folds estimation is obtained when we used the LOOCV method (during raw cross-validation and then during adjusted cross-validation). The significant codes (0.1, 0.01, 0.001, and 0.05) with minimum deviance residuals are assessed (Table 3).

**Table 2:** List of 50-differentially expressed genes.

**S. no**	**Probe ID**	**Gene ID**	**logFC**	**t**	***P*-value**	**adj.P.Val**	**B**	**Aberration**
1	240287_at	irg1	−5.93568	−50.392	7.88E-12	2.30E-07	14.35412	Down-regulated
2	235737_at	TSLP	−5.81414	−50.0105	8.41E-12	2.30E-07	14.33257	
3	235574_at	GBP4	−4.36184	−40.5895	4.93E-11	8.98E-07	13.64744	
4	205207_at	IL6	−5.18603	−37.197	1.03E-10	1.41E-06	13.30453	
5	202510_s_at	TNFAIP2	−4.1804	−34.7333	1.84E-10	2.01E-06	13.01096	
6	202269_x_at	GBP1	−3.20432	−33.4148	2.55E-10	2.32E-06	12.83562	
7	227458_at	CD274	−5.57009	−31.9417	3.73E-10	2.91E-06	12.62265	
8	223501_at	TNFSF13B	−4.88682	−31.3969	4.31E-10	2.95E-06	12.53892	
9	210797_s_at	OASL	−4.56379	−29.9747	6.38E-10	3.87E-06	12.3066	
10	214038_at	ccl8	−6.57392	−28.3084	1.03E-09	5.65E-06	12.00654	
11	205013_s_at	Adora2a	−3.81754	−27.4662	1.33E-09	6.62E-06	11.84223	
12	205569_at	LAMP3	−6.29062	−26.5359	1.78E-09	8.03E-06	11.64986	
13	229450_at	Ifit3	−4.28387	−26.3161	1.91E-09	8.03E-06	11.60267	
14	222793_at	DDX58	−4.25905	−25.5566	2.44E-09	8.71E-06	11.43411	
15	218810_at	ZC3H12A	−2.84809	−25.299	2.66E-09	8.71E-06	11.37495	
16	205660_at	OASL	−4.97761	−25.0953	2.84E-09	8.71E-06	11.32745	
17	202688_at	TNFSF10	−5.15206	−25.0718	2.87E-09	8.71E-06	11.32192	
18	205599_at	Traf1	−4.14304	−24.8005	3.14E-09	8.87E-06	11.2575	
19	204747_at	Ifit3	−4.25172	−24.7046	3.25E-09	8.87E-06	11.23446	
20	219716_at	APOL6	−2.70983	−24.2599	3.78E-09	9.53E-06	11.12554	
21	202759_s_at	akap2/PALM2	−2.47769	−24.2173	3.84E-09	9.53E-06	11.11493	
22	230036_at	SAMD9L	−2.94113	−22.691	6.62E-09	1.57E-05	10.71328	
23	235643_at	SAMD9L	−3.19263	−22.0591	8.39E-09	1.84E-05	10.53392	
24	223502_s_at	TNFSF13B	−5.13985	−21.8857	8.96E-09	1.84E-05	10.48325	
25	205992_s_at	IL15	−4.10568	−21.8596	9.05E-09	1.84E-05	10.47556	
26	209038_s_at	EHD1	−3.57268	−21.8496	9.09E-09	1.84E-05	10.47261	
27	209037_s_at	EHD1	−4.10382	−21.4847	1.05E-08	2.04E-05	10.36366	
28	242752_at	–	−2.33834	−21.2349	1.15E-08	2.08E-05	10.28736	
29	226757_at	Ifit2	−5.22852	−21.224	1.16E-08	2.08E-05	10.28402	
30	231577_s_at	GBP1	−2.98548	−21.1802	1.18E-08	2.08E-05	10.27046	
31	206157_at	Ptx3	−3.72465	−20.8941	1.32E-08	2.19E-05	10.18102	
32	208012_x_at	SP110	−2.34041	−20.8471	1.35E-08	2.19E-05	10.16617	
33	202760_s_at	akap2/PALM2	−3.0481	−20.8187	1.36E-08	2.19E-05	10.15714	
34	218943_s_at	DDX58	−4.97703	−20.6726	1.44E-08	2.25E-05	10.11048	
35	202086_at	mx1	−3.63011	−19.9264	1.96E-08	2.98E-05	9.864092	
36	225344_at	NCOA7	−2.6496	−19.8461	2.03E-08	3.00E-05	9.836755	
37	215495_s_at	samd4a	−1.78695	−19.4961	2.35E-08	3.39E-05	9.715643	
38	220104_at	ZC3HAV1	−3.4764	−19.3094	2.55E-08	3.57E-05	9.649722	
39	210029_at	IDO1	−6.46305	−19.2265	2.64E-08	3.61E-05	9.620158	
40	211122_s_at	CXCL11	−7.63526	−19.0851	2.81E-08	3.75E-05	9.569288	
41	1557905_s_at	CD44	−2.72423	−18.8999	3.05E-08	3.88E-05	9.501801	
42	1555464_at	IFIH1	−4.11661	−18.8975	3.05E-08	3.88E-05	9.500928	
43	229221_at	CD44	−3.80452	−18.6478	3.41E-08	4.16E-05	9.408394	
44	210163_at	CXCL11	−6.82817	−18.6392	3.42E-08	4.16E-05	9.405188	
45	209723_at	SERPINB9	−4.33364	−18.5564	3.55E-08	4.22E-05	9.37409	
46	204103_at	CCL4	−4.92524	−18.3405	3.91E-08	4.55E-05	9.292083	
47	214329_x_at	TNFSF10	−5.31551	−18.2625	4.05E-08	4.57E-05	9.262101	
48	203915_at	Cxcl9	−4.43103	−18.2429	4.09E-08	4.57E-05	9.254554	
49	210285_x_at	WTAP	−2.59583	−18.1602	4.25E-08	4.65E-05	9.22254	
50	220675_s_at	pnpla3	2.423905	25.63874	2.38E-09	8.71E-06	11.45275	Up-regulated

**Table 3:** k-fold cross validation by bioconductor “boot” package using dispersion parameter of Gaussian family.

	**Estimate**	**Std. Error**	***t*-value**	**Pr(>|t|)**
(Intercept)	0.023823	0.003194	7.46^*^	8.81E^−14^^***^
x1	0.349441	0.00554	63.071	<2.00E^−16^^***^
x2	0.078029	0.00486	16.056	<2.00E^−16^^***^
x3	0.150248	0.002554	58.83	<2.00E^−16^^***^
x4	−0.09253	0.00321	−28.827	<2.00E^−16^^***^
x5	−0.00143	0.002231	−0.639^**^	0.523^*^
x6	0.773287	0.002425	318.819	<2.00E^−16^^***^
x7	−0.22529	0.005723	−39.362	<2.00E^−16^^***^
x8	−0.03568	0.00451	−7.91	<2.62E^−15^^***^

*Deviance Residuals: Min (−3.1894), 1Q (−0.0996), Median (0.0004), 3Q (0.1016), Max (1.2137)*.

Signif. codes: 0

“***” 0.001

“**” 0.01

“*” *0.05 “.” 0.1 “ ” 1*.

*Number of Fisher Scoring iterations: 2*.

*Null deviance: 195495.4 on 54674 degrees of freedom*.

*Residual deviance: 1786.6 on 54666 degrees of freedom*.

### Identifying cancer-related genes

Among differentially expressed genes, 7-cancer related genes were obtained including TSLP, IL6, GBP1, CD274, TNFSF13B, OASL, and pnpla3 after mapping (*p* < 0.00005) with OMIM and Cancer Genetics Web database. The references to the role of these genes in cancer have been shown in Table 4 using PubMed database.

**Table 4:** The differentially expressed cancer-related genes curated from OMIM and Cancer Genetics. Web databases and their role has been referenced from PubMed.

**S. no**	**Probe ID**	**Gene ID**	**Uniprot_ID**	**PubMed count**	**Protein name**	**Reference links**
1	235737_at	TSLP	TSLP_HUMAN	68	Thymic stromal lymphopoietin	http://www.ncbi.nlm.nih.gov/pubmed/?term=%E2%80%9CTSLP%E2%80%9D+and+%E2%80%9CCancer%E2%80%9D
2	205207_at	IL6	IL6_HUMAN	1067	Interleukin-6	http://www.ncbi.nlm.nih.gov/pubmed/?term=%E2%80%9CIL6%E2%80%9D+and+%E2%80%9CCancer%E2%80%9D
3	202269_x_at	GBP1	GBP1_HUMAN	26	Interferon-induced guanylate-binding protein	http://www.ncbi.nlm.nih.gov/pubmed/?term=%E2%80%9CGBP1%E2%80%9D+and+%E2%80%9CCancer%E2%80%9D
4	227458_at	CD274	PD1L1_HUMAN	430	Programmed cell death-1	http://www.ncbi.nlm.nih.gov/pubmed/?term=%E2%80%9CCD274%E2%80%9D+and+%E2%80%9CCancer%E2%80%9D
5	223501_at	TNFSF13B	TN13B_HUMAN	68	Tumor necrosis factor ligand superfamily member 13B	http://www.ncbi.nlm.nih.gov/pubmed/?term=%E2%80%9CTNFSF13B%E2%80%9D+and+%E2%80%9CCancer%E2%80%9D
6	210797_s_at	OASL	OASL_HUMAN	12	2′-5′-oligoadenylate synthase	http://www.ncbi.nlm.nih.gov/pubmed/?term=%E2%80%9COASL%E2%80%9D+and+%E2%80%9CCancer%E2%80%9D
7	220675_s_at	pnpla3	PLPL3_HUMAN	27	Patatin-like phospholipase domain-c	http://www.ncbi.nlm.nih.gov/pubmed/?term=%E2%80%9Cpnpla3%E2%80%9D+and+%E2%80%9CCancer%E2%80%9D

### Cluster and functional enrichment analysis of DEGs

**Figure 3:**
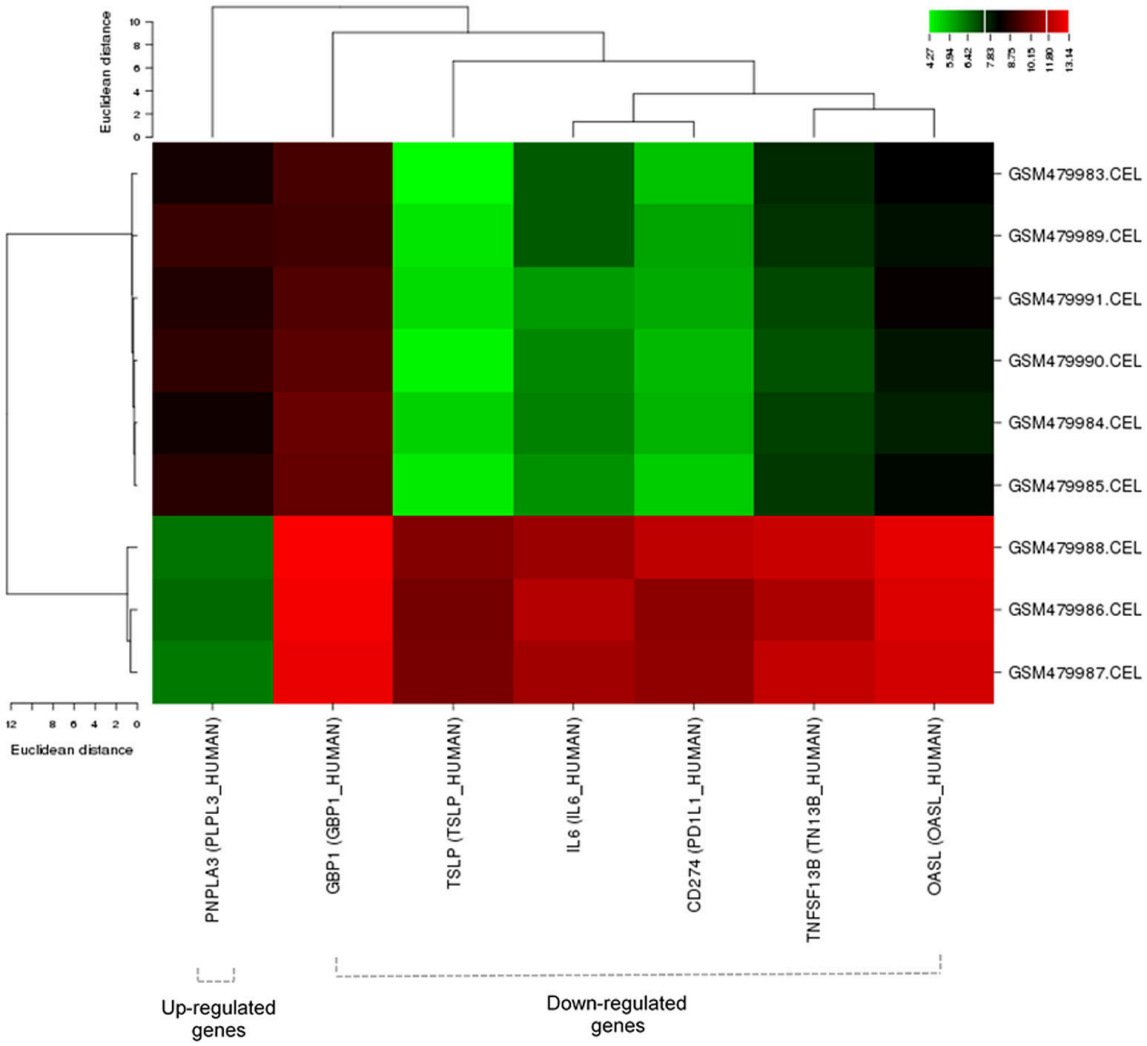
Cluster analysis of 7 cancer-related DEGs with Euclidean distance (Binning method: Quantile). Green corresponds to a small distance and Red to a large distance. Lines indicate the boundaries of the clusters in the level of the tree. Dotted lines indicate down or up-regulated genes.

**Figure 4:**
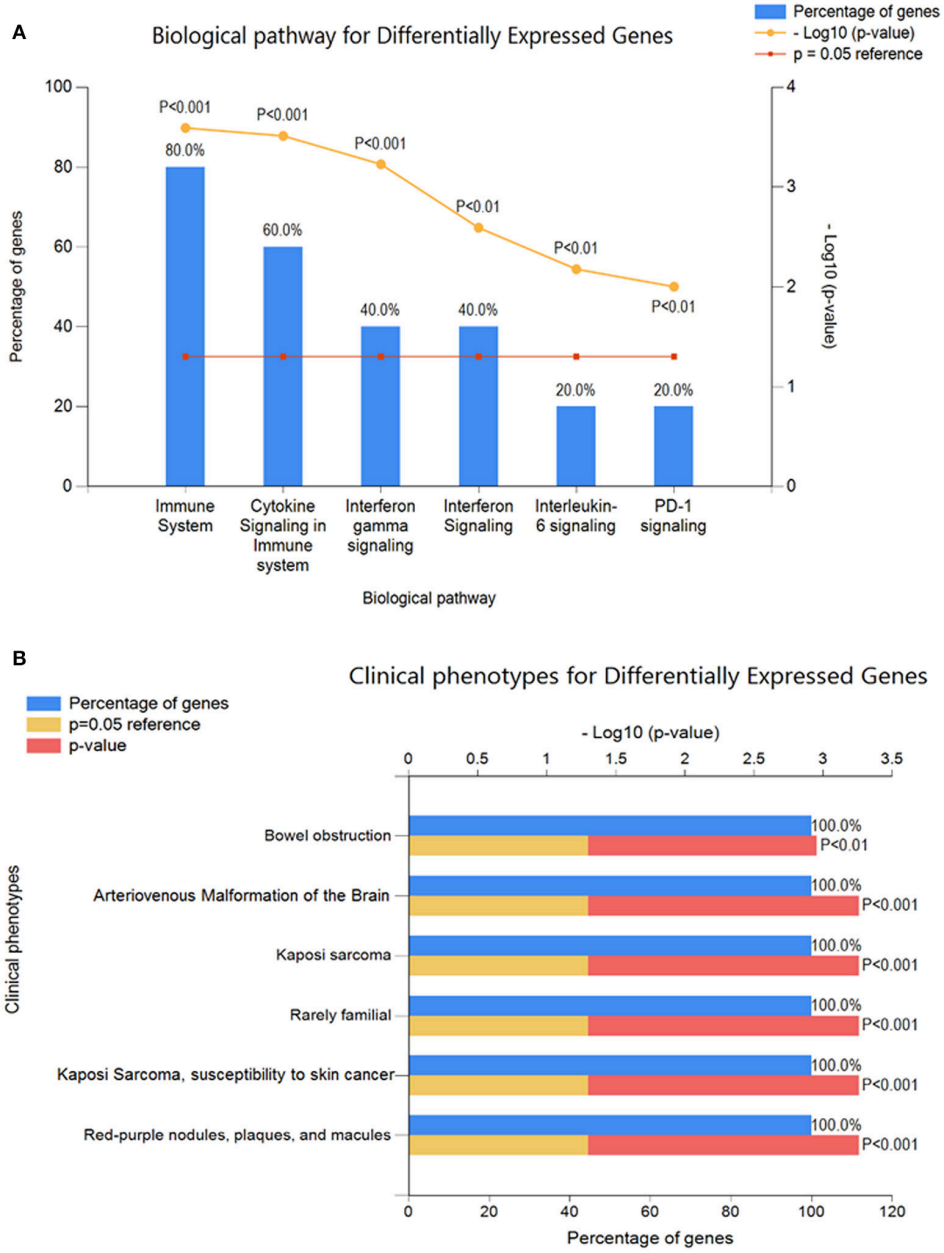
Functional enrichment analysis of expressively cancer-related DEGs using FunRich annotation tool **(A)** Biological pathways analysis **(B)** clinical phenotypes for GEGs.

Clustering analysis has recognized to be helpful to understand gene function, gene regulation, cellular processes, and subtypes of cells. The cluster analysis of significantly expressed 7-cancer related genes was studied with Euclidean distance (Figure 3). The genetic expression of Shiga effected cell samples is distinguished from the untreated replicates, indicating that obvious differences existed between the two groups (treated and untreated). The analysis showed that 6 down-regulated and 1 up-regulated genes were significantly enriched. The total number of enriched terms was calculated with their significant cutoff parameters (Table 5). The immune response (*p* < 1.68E-05), regulation of lymphocyte's proliferation (*p* < 0.001017), T-cell activation (p < 0.002007), cytokine activity (p < 0.004485), leukocyte homeostasis (*p* < 0.021097) and patatin-like phospholipase domain containing-3 were expressively enriched among dysregulated genes. These gene products are related to immune response system and signaling pathways, signaling of chemical mediators and regulation of a cell defense system (Figure 4A). The dysregulation of these genes has been found associated with Kaposi sarcoma, cerebral malformation, familial polyposis, and other clinical phenotypes (Figure 4B).

**Table 5:** Functional and GO^*^ analysis of 7-differentially expressed genes.

**S.no**	**Term**	**Count**	***P*-value**	**Fold enrichment**
1	[GO:0006955] immune response	6	1.68E-05	13.1
2	[GO:0042129] regulation of T cell proliferation	3	5.69E-04	72.7
3	[GO:0050670] regulation of lymphocyte proliferation	3	0.001017	54.3
4	[GO:0070663] regulation of leukocyte proliferation	3	0.001041	53.7
5	[GO:0032944] regulation of mononuclear cell proliferation	3	0.001041	53.7
6	[GO:0050863] regulation of T cell activation	3	0.002007	38.5
7	[GO:0051249] regulation of lymphocyte activation	3	0.003188	30.5
8	[GO:0002694] regulation of leukocyte activation	3	0.003992	27.2
9	[GO:0050865] regulation of cell activation	3	0.004426	25.8
10	[GO:0005125] cytokine activity	3	0.004485	25.0
11	[GO:0050871] positive regulation of B cell activation	2	0.019354	91.1
12	[GO:0001776] leukocyte homeostasis	2	0.021097	83.5
13	[GO:0042102] positive regulation of T cell proliferation	2	0.022838	77.1
14	[GO:0050864] regulation of B cell activation	2	0.029772	58.9
15	[GO:0050671] positive regulation of lymphocyte proliferation	2	0.032074	54.7
16	[GO:0032946] positive regulation of mononuclear cell proliferation	2	0.032649	53.7
17	[GO:0070665] positive regulation of leukocyte proliferation	2	0.032649	53.7
18	[GO:0050870] positive regulation of T cell activation	2	0.044081	39.6
19	[GO:0002237] response to molecule of bacterial origin	2	0.049753	35.0
20	[GO:0051251] positive regulation of lymphocyte activation	2	0.055958	31.0
21	[GO:0048872] homeostasis of number of cells	2	0.057644	30.1
22	[GO:0002696] positive regulation of leukocyte activation	2	0.061008	28.4
23	[GO:0050867] positive regulation of cell activation	2	0.063803	27.1
24	[GO:0042127] regulation of cell proliferation	3	0.074923	5.7
25	[GO:0002252] immune effector process	2	0.076569	22.4

* *GO, Gene Ontology*.

### Protein-protein interaction network

**Figure 5:**
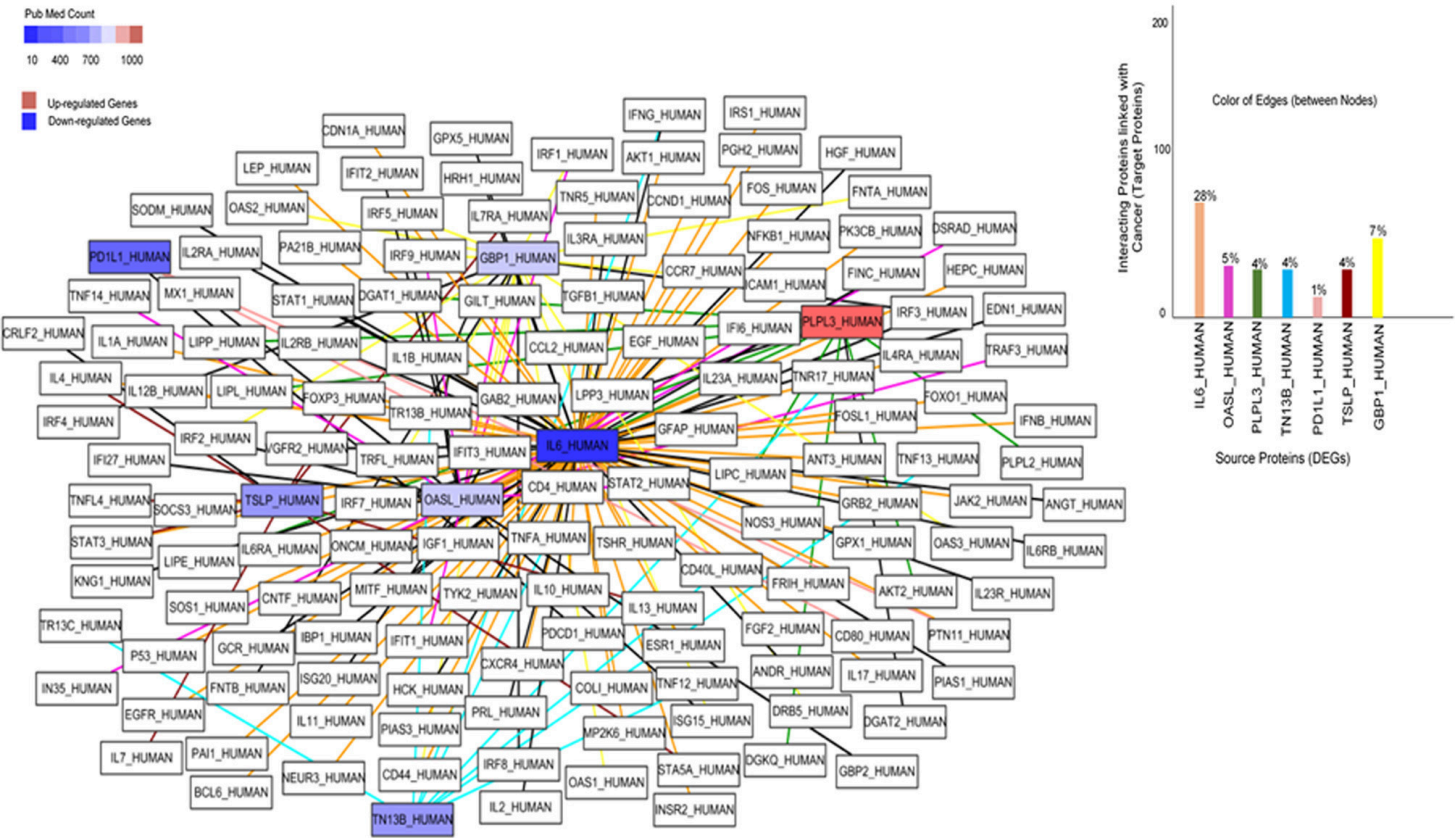
Protein-Protein Interaction network of cancer-related differential expressed genes. Each node represents the protein, while lines indicate the interaction edges. The association of these source proteins and their interactors (target proteins) in cancers are shown in a bar graph. Blue and red color indicate down and up-regulated genes, respectively.

In PPI network, a total of 215 nodes was retrieved from STRING and HAPPI databases. In this network, IL6_Human as a source protein is connected with a number of target proteins (interactors) (28%) that have been reported in several cancers followed by the PD1L1_HUMAN (7%) (Figure 5). Overexpression of PLPL3_HUMAN is associated with 4% of interactors that are responsible for various types of cancers.

**Figure 6:**
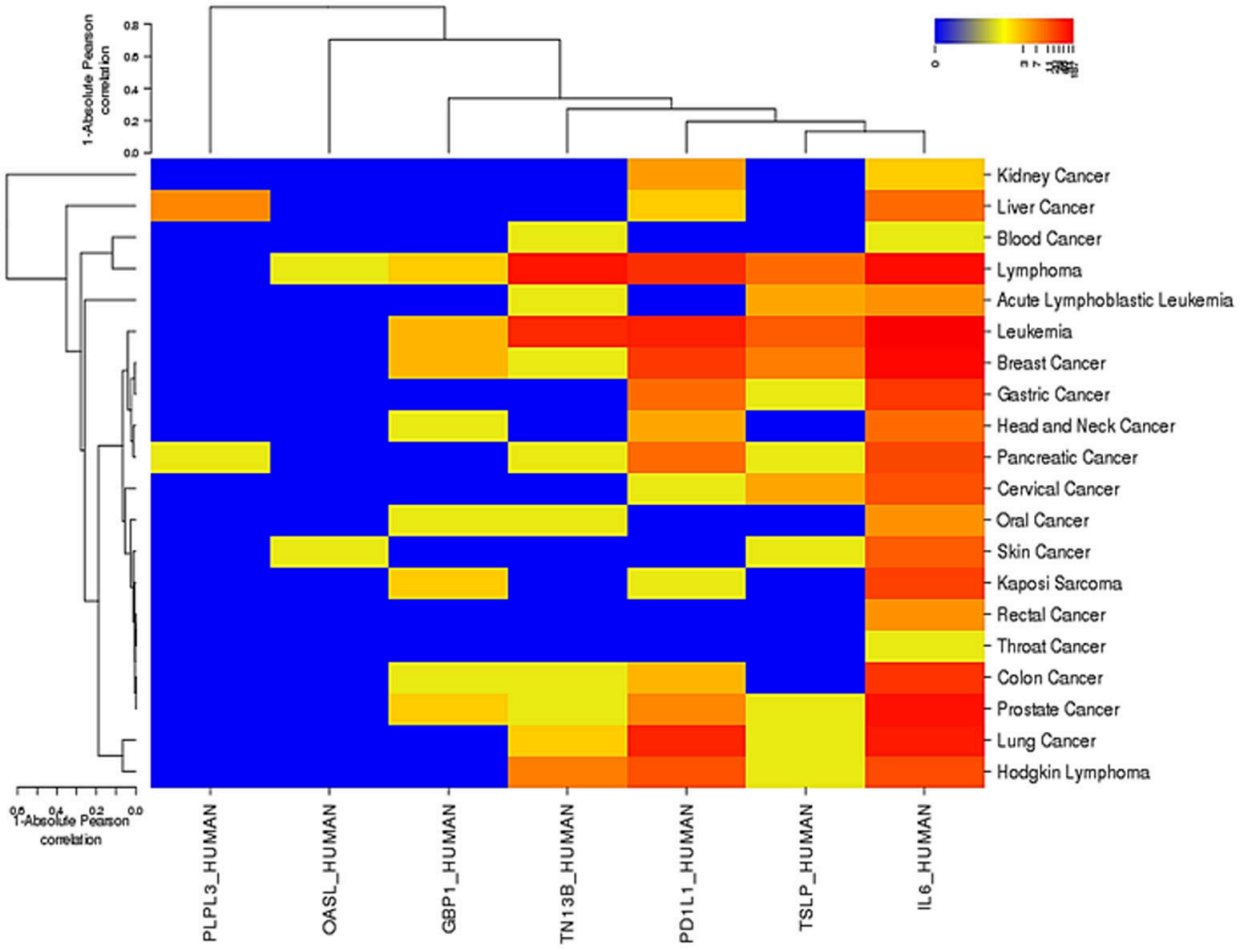
The role of 7 DEGs in various cancers with Absolute Pearson correlation. Red color indicates the maximum involvement of a gene in various cancers and Blue color represents the least association. This association was curated using OMIM, Cancer Genomic Web, and PubMed databases.

It has been shown that IL6_HUMAN is involved in the progression of several cancers, extremely responsible for leukemia, breast cancer and lymphoma (Figure 6). PD1L1_HUMAN has also been found the connection in a number of cancer cases, including lung cancer, leukemia, Hodgkin lymphoma, and head/neck cancers. The observed least association of OASL_HUMAN is with lymphoma and skin cancer cases.

### *De novo* prediction of regulatory motifs

**Figure 7:**
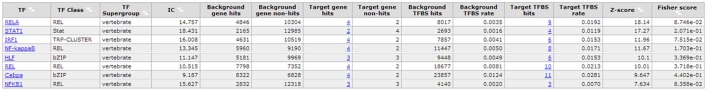
Over-representation of cancer-related DEGs using oPOSSUM with 80% Matrix score.

Cancer-related differentially expressed genes were used for over-representation and de novo analysis. Following transcription factors (TF) class were found in these DEGs: REL, Stat, TRP-CLUSTER, and bZIP. They are related to six target matrices in the Jaspar core profile. Opossum-tool found RELA as a top-ranked matrix under both the Fisher-score (8.746e^−02^) and the z-score (18.14) followed by the STAT1 which satisfied the thresholds for significant over-representation (Figure 7). This shows that genes containing conserved RELA, STATI, IRFI, NF-kappaB, PEND, HLF, REL, CEBPA, DI_2, and NFKB1 binding sites (Supplementary Figure 2) are indeed over-represented in cancer-related DEGs and this over-representation can be recuperated computationally. The rank of parameter settings for the analysis of oPOSSUM-tool could reflect properties how a specific TF controls its targets.

## Discussion

Monocytes/macrophages are key cellular components of the human immune system. Upon exposure to Shiga toxins produced by *Shigella dysenteriae* serotype 1 and enterohaemorrhagic *Escherichia coli*, these cells secrete chemical mediators, which initiate in?ammation and activate of immune system signals (Harrison et al., 2005). It has been known that Stx alters the genetic expression and several signaling pathways triggered by the toxin modify the expression of several genes in mammalian cells. The comparative cDNA studies showed that Stx toxins modulated the transcriptional response of human macrophage-like THP-1 cells (Leyva-Illades et al., 2010) associated with a number of abnormalities. In another study, it was observed that Stxs dysregulated the expression of Gb3/CD77 of lung carcinoma cell lines (Uchida et al., 1999). Although it is known that these toxins alter the genetic expression and affect the cellular division and apoptosis pathway (Elmore, 2007), the underlying mechanisms and its association with cancer development remain unclear. The purpose of the current study was to investigate differentially expressed genes of human THP1 cells in response to Stxs and the association of these genes with cancer development.

In this study, we found 50 DEGs, and 49 of which were down-regulated and only a single gene being up-regulated by Stxs. Among these, 7-genes were mapped with cancer development that fell into the following functional categories: Immune response, regulation of lymphocyte's proliferation, cytokine activity and patatin-like phospholipase domain containing-3. These DEGs are linked with important biological pathways that are related to immune response signaling pathways, signaling of chemical mediators and regulation of cell defense system. Dysregulation of these genes can cause Kaposi sarcoma, familial polyposis, and other clinical phenotypes. These findings appear different from earlier microarray analyses performed by Keepers et al. (2006), which revealed Stxs-encoded proteins with functions related to transcriptional regulation, cell proliferation, and cell cycle regulation. Cell proliferation, cell morphogenesis, immune response signaling, anti-apoptosis, and regulation of cell death are strongly associated with the down-regulated genes, whereas cell cycle process, glycerolipid metabolism, and Patatin-like phospholipase activity are enriched in the up-regulated genes. These factors are known to cancer progression (Hunter and Pines, 1994; DeRisi et al., 1996).

Stxs have been reported to up-regulate the expression of cytokines and chemokines from human primary blood monocytes and to transform monocytic cell lines *in vitro*, and to affect cytokine expression via several mechanisms, including the activation of MAPK cascades leading to increased cytokine gene transcriptional activity and improved translation initiation efficiency (Harrison et al., 2005). However, in our analysis, we found that Stxs down-regulated IL6 gene, which is a potent inducer of the acute phase response and plays an important role in the final differentiation of B-cells into Ig-secreting cells responsible for lymphocyte and monocyte differentiation (Hirano et al., 1986). The downregulation of IL-6 and other DEGs is associated with highly malignant mammary carcinomas (Fontanini et al., 1999). Some genes such as MMP11 (extra-cellular matrix proteins), XRCC1 (DNA repair), VEGF (regulator of angiogenesis), Cyclin D1 (cell cycle regulators) and tumor-suppressor genes (Semaphorin 3B, WNT-5A) have been found as down-regulated genes during lung cancer progression (Campioni et al., 2008). Recently it has studied that Stxs elicit a proinflammatory response, including the elevated production of TNF-α, IL-1β, IL-8, IL-6, and growth-related oncogene α (Groα) both *in vitro* and *in vivo* (Lee et al., 2013). Furthermore, our analysis reveals that toxin may affect IL6-expression via a number of mechanisms, including JAK-STAT cascade, cytosolic DNA-sensing, TNF signaling pathway and transcriptional misregulation in cancer cells. IL6 is associated with the progression of multiple cancers, including leukemia, lymphoma, breast cancer, kidney, and lungs cancer. TNFSF13B encodes Tumor Necrosis Factor (Ligand) Superfamily, Member 13b, also referred to as B-Cell-Activating Factor. It is important in the proliferation and differentiation of B-cells that can act as a transcription factor for its own gene in association with NF-κB p50 (Yu et al., 2000). Similarly, CD274 is another gene that is involved in the co-stimulatory signal, essential for T-cell proliferation and production of IL10 and IFNG. The dysregulation of this gene inhibits T-cell proliferation and cytokine production causing a number of cancer including lung, leukemia, Hodgkin lymphoma, and head/neck cancers (Dong et al., 1999). We found cytokines and chemokines, including IL-8, CSF2, GRO-1, GRO-2, and GRO-3, are affected by toxin exposure (Leyva-Illades et al., 2010). In addition, expression of TSLP, IL6, GBP1, CD274, TNFSF13B, OASL, and pnpla3 involved in the immune system, cell cycle, T-cell activation and lymphocyte's proliferation were also altered in THP1 cells by toxin treatment. Dysregulation of these genes cause increased apoptosis, cellular division and cancerous activities (Dong et al., 1999; Andreoli et al., 2014; Barooei et al., 2015; Hengeveld and Kersten, 2015; Ibsen et al., 2015; Manda et al., 2015; Ali et al., 2016). We performed motif enrichment to predict the transcription factor binding sites and over-represented regions of these cancer-related genes to better understand the complex biological mechanism. Several motifs, including RELA, STATI, IRFI, NF-kappaB, PEND, HLF, REL, CEBPA, DI_2, and NFKB1 were found in the gene set for factors that play critical roles in modeling required for immune response system, cellular cycles, chemical mediation and cell growth.

It should be noted that the publicly accessible microarray dataset studied here were subjected to rigorous statistical algorithms. Thus, there is a high level of confidence in the list of differentially expressed genes altered by Stxs. The present study did not evaluate an independent prognostic factor but suggest that differentially expressed genes should be evaluated as potential biomarkers for further study to uncover their possible effects in the diagnosis, treatment, and prognosis of cancers.

## Conclusion

This study identified differentially expressed genes in human THP1-monocytic cells upon treatment by bacterial Shiga toxins, and many of these genes are involved in the pathophysiological carcinomas. Stxs not only inhibited the protein synthesis of target cells but even induce the genetic expression and activate the multiple signaling pathways. Thus, cells treated with Stxs may lead to progression of multiple cancers. These findings may provide a valuable framework for developing diagnostic biomarkers and treatment strategies for cancer. Further molecular and real-time studies are warranted to investigate the role of the identified genetic elements in cancer development.

## Author contributions

SM and JG performed the research and wrote the paper; TN and XW analyzed the data; BB and XY collected data and proofread the article; JC supervised and helped perform the research and commented on the draft manuscript.

### Conflict of interest statement

The authors declare that the research was conducted in the absence of any commercial or financial relationships that could be construed as a potential conflict of interest.

## Footnotes

^1^Gene Expression Omnibus: http://www.ncbi.nlm.nih.gov/geo/. Accessed 2016-05.25.

^2^NCBI: http://www.ncbi.nlm.nih.gov/omim. Accessed 2015-08.28.

^3^Cancer Genetics Web: http://www.cancer-genetics.org/index.htm. Accessed at Thursday Sep 10, 2016.

^4^PubMed: http://www.ncbi.nlm.nih.gov/pubmed. Accessed 2015-08-28.

^5^National Cancer Database: http://www.facs.org. Accessed 2016-01.01.

## Supplementary material

Supplementary Figure 1 Framework our study indicates the effects of Shiga toxins on cancer progressions.

Supplementary Figure 2 Transcription factor binding sites (TFBS) of cancer-related differentially expressed genes with their Jaspar core profiles. Top 10% of conserved regions (min. conservation 70%) are using oPOSSUM tool.
