# Supplementary figures and images for: Simulation Study of cDNA Dataset to Investigate Possible Association of Differentially Expressed Genes of Human THP1-Monocytic Cells in Cancer Progression Affected by Bacterial Shiga Toxins
Source: Front Microbiol. 2018 Mar 13;9:380. doi: 10.3389/fmicb.2018.00380 (PMC5859033; doi:10.3389/fmicb.2018.00380)

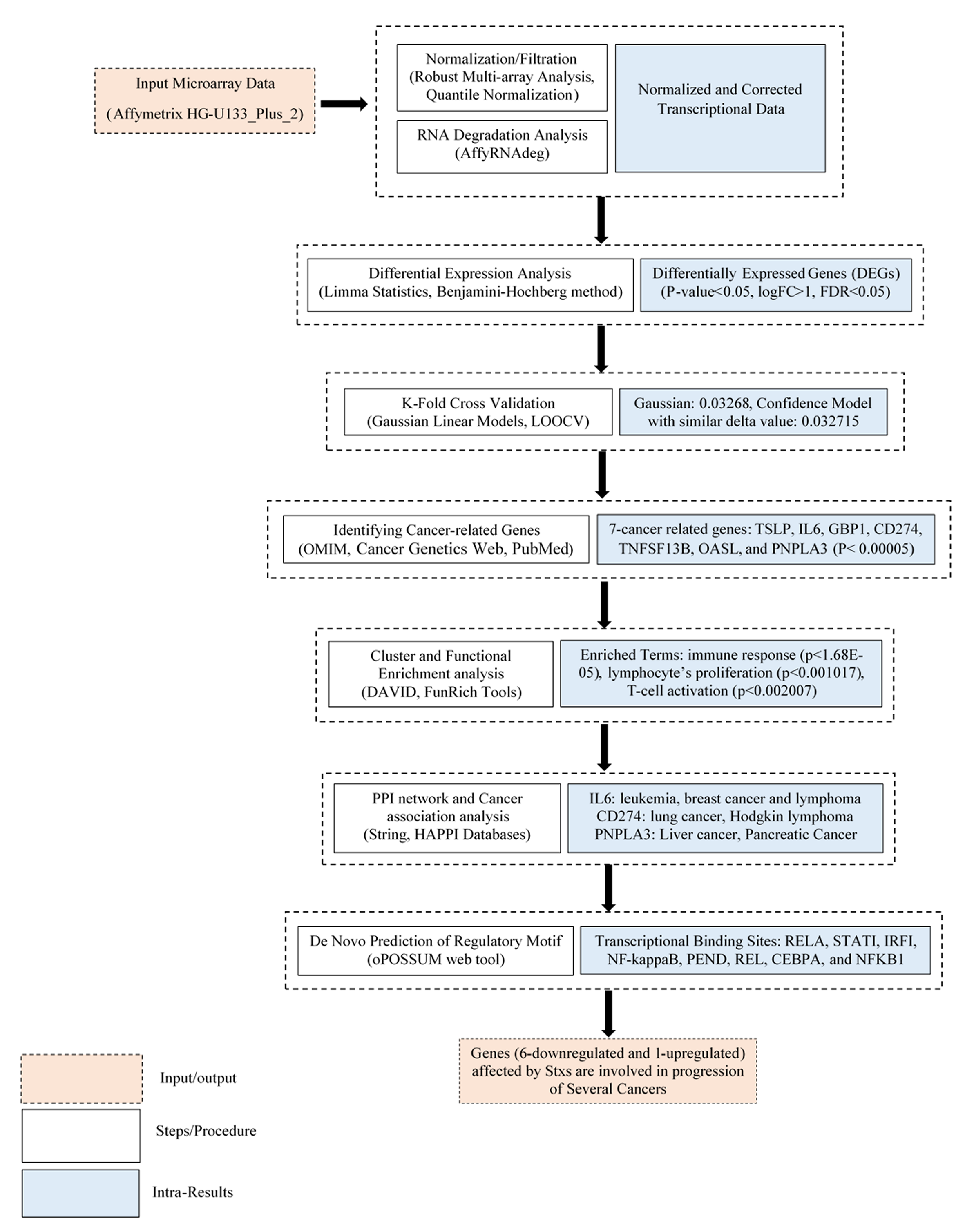

Supplement: Supplementary Figure 1 — Framework our study indicates the effects of Shiga toxins on cancer progressions. [file Image1.TIF]

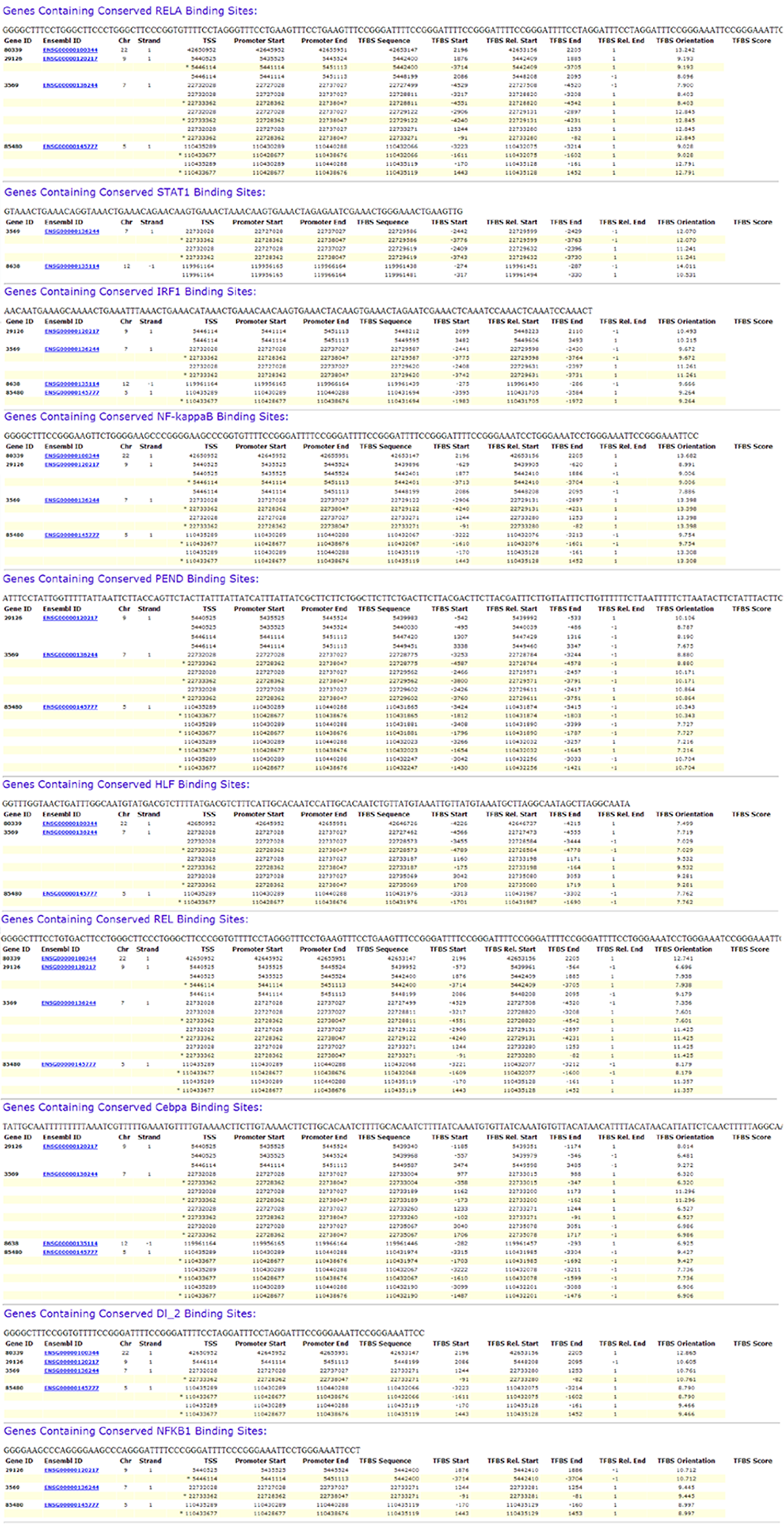

Supplement: Supplementary Figure 2 — Transcription factor binding sites (TFBS) of cancer-related differentially expressed genes with their Jaspar core profiles. Top 10% of conserved regions (min. conservation 70%) are using oPOSSUM tool. [file Image2.TIF]
